# TMEM59 deficiency activates chaperone‐mediated autophagy and ameliorates disease‐like pathologies in tauopathy model mice

**DOI:** 10.1002/alz.70369

**Published:** 2025-06-23

**Authors:** Naizhen Zheng, Zijie Wang, Jing Cao, Kun Li, Hui Xu, Jinghui Wang, Lingliang Zhang, Jian Meng, Ziqian Tang, Hong Luo, Hao Sun, Xian Zhang, Yun‐wu Zhang

**Affiliations:** ^1^ Xiamen Key Laboratory of Brain Center, The First Affiliated Hospital of Xiamen University, and Fujian Provincial Key Laboratory of Neurodegenerative Disease and Aging Research, Institute of Neuroscience, School of Medicine Xiamen University Xiamen China; ^2^ State Key Laboratory of Cellular Stress Biology School of Life Sciences Xiamen University Xiamen China

**Keywords:** Alzheimer's disease, chaperone‐mediated autophagy, heat‐shock cognate 71 kDa, lysosome‐associated membrane protein type 2A, tau, tauopathy, transmembrane protein 59

## Abstract

**INTRODUCTION:**

Tauopathy is characterized by the pathology of tau deposits in the brain. Transmembrane protein 59 (TMEM59) is correlated with Alzheimer's disease (AD), the most common type of tauopathy. However, whether and how TMEM59 regulates tau pathology remains unknown.

**METHODS:**

We analyzed TMEM59 levels in the brains of AD patients and the tau^P301S^ transgenic (PS19) mice, evaluated behaviors and tauopathy‐related pathologies in PS19 mice with TMEM59 haploinsufficiency, and studied the regulation of TMEM59 on chaperone‐mediated autophagy (CMA) using biochemical analysis.

**RESULTS:**

TMEM59 levels increased in the brains of AD patients and PS19 mice at pathological stages. TMEM59 haploinsufficiency attenuated cognitive deficits and disease‐related pathologies in PS19 mice. TMEM59 deficiency promoted lysosome‐associated membrane protein type 2A levels and CMA activity, whereas TMEM59 overexpression had the opposite effects.

**DISCUSSION:**

Our study identifies an important role of TMEM59 in regulating CMA and reveals the potential of targeting TMEM59 for tauopathy intervention.

**Highlights:**

- Transmembrane protein 59 (TMEM59) levels increase in the brains of Alzheimer's disease patients and the tau^P301S^ transgenic (PS19) tauopathy model mice at pathological stages.
- TMEM59 haploinsufficiency attenuates cognitive deficits, neurodegeneration, synapse dysfunction, gliosis, neuroinflammation, and tau pathology in PS19 mice.
- TMEM59 interacts with lysosome‐associated membrane protein type 2A and heat‐shock cognate 71 kDa and regulates chaperone‐mediated autophagy.
- TMEM59 may serve as a therapeutic target for tauopathy.

## 1 BACKGROUND

Tauopathy is a group of neurodegenerative diseases that are clinically heterogeneous but share a common pathology of tau deposits in the brain.^1^, ^2^ Tau is a microtubule‐associated protein that stabilizes neuronal microtubules and thus promotes axonal outgrowth under physiological conditions.^3^ However, abnormal post‐translational modifications of tau, especially hyperphosphorylation, can result in tau misfolding and aggregation.^1^, ^2^, ^3^, ^4^ Tau pathology can lead to synaptic loss, neuroinflammation, and cognitive decline, resulting in the pathogenesis of various tauopathic disorders, such as Alzheimer's disease (AD), progressive supranuclear palsy, corticobasal degeneration, corticobasal degeneration nucleus, and argyrophilic granular disease.

Chaperone‐mediated autophagy (CMA), a lysosome‐dependent selective degradation pathway, contributes to the degradation of neurodegeneration‐related proteins such as α‐synuclein and tau.^5^, ^6^, ^7^ CMA is triggered when specific cytosolic proteins bearing a targeting motif (KFERQ‐like motif) are recognized by a chaperone protein, heat‐shock cognate 71 kDa (HSC70). HSC70 delivers the substrate to the lysosome surface and binds to the lysosomal receptor lysosome‐associated membrane protein type 2A (LAMP2A), thereby triggering LAMP2A multimerization into a complex to mediate the translation of the substrate into the lysosomal lumen for degradation.^5^, ^8^ LAMP2A is the rate‐limiting component of CMA, and changes in levels and dynamics of LAMP2A at the lysosomal membrane affect CMA flux.^5^, ^8^ Several previous studies have shown that LAMP2A deficiency deteriorates tau pathologies in AD model mice,^5^, ^8^ whereas pathologic tau can interfere with the CMA pathway and compromise CMA‐mediated pathologic tau degradation.^7^, ^9^ Nevertheless, the detailed molecular mechanism underlying CMA and its involvement in tauopathy and other neurodegenerative diseases requires further investigation.

Transmembrane protein 59 (TMEM59), also known as dendritic cell factor 1 (DCF1), is a macroautophagy‐related type I transmembrane protein.^10^, ^11^, ^12^ TMEM59 can interact with ATG16L1 to promote LC3 activation and induce macroautophagy.^10^, ^11^ Our previous study, as well as others, have found that TMEM59 deficiency can attenuate amyloid beta (Aβ) aggregation and cognitive deficits in animal models resembling Aβ pathology in AD.^13^, ^14^ However, whether and how TMEM59 affects tau pathology in AD and other tauopathic disorders has yet to be determined.

Herein, we find that TMEM59 protein levels increase in the brains of AD patients and the tau^P301S^ transgenic (PS19) tauopathy mouse model at pathological stages. Moreover, we show that TMEM59 haploinsufficiency attenuates cognitive deficits and disease‐related pathologies in PS19 mice. Mechanistically, we reveal that TMEM59 deficiency promotes LAMP2A protein levels and CMA activity, whereas TMEM59 overexpression has the opposite effects. Therefore, our study demonstrates that TMEM59 deficiency can attenuate disease‐like phenotypes in PS19 mice through activating CMA.

## 2 METHODS

### 2.1 Human samples

Human brain tissue lysates were from National Developmental and Functional Human Brain Bank, Chinese Academy of Medical Sciences, National Health and Disease Human Brain Tissue Resource Center, and Brain Bank and Neurodegenerative Disease Research Center in China. The primary neuropathologic diagnosis, age at death, and sex of the human subjects are listed in Table S1 in supporting information.

### 2.2 Animals and ethics statement

*Tmem59^+/−^* mice were generated as previously described.^15^ PS19 mice (IMSR_JAX:008169) were obtained from Jackson Laboratory. These mice were maintained and bred at Xiamen University Laboratory Animal Center. All animal procedures were performed according to the guidelines of the National Institutional Animal Health Guide for the Care and Use of Laboratory Animals and approved by the animal ethics committee of Xiamen University (XMULAC20170209).

### 2.3 RNA interference

Small interfering RNAs (siRNAs) were synthesized by GenePharma. The human *TMEM59* targeting siRNA sequence used is 5′‐GCACAGAGCUUCAUAACCU‐3′. The negative control siRNA sequence used is 5′‐UUCUCCGAACGUGUCACGU‐3′. siRNAs were transfected into HERK293T using Entranster‐R4000 reagent (Engreen), following the manufacturer's instructions.

### 2.4 Cell culture and transfection

HEK293T cells were cultured in Dulbecco's Modified Eagle Medium containing 10% fetal bovine serum. Plasmids were transfected into HEK293T cells using TurboFect Transfection Reagent (Thermo Fisher Scientific), following the manufacturer's instructions.

Primary neurons were prepared from the cerebral cortex and hippocampus of mice at embryonic day (E) 16.5 and cultured in neurobasal medium (Thermo Fisher Scientific) supplied with 2% B27 (Thermo Fisher Scientific) and 1 mM glutamine (Thermo Fisher Scientific). Primary neurons were transfected with plasmids using Lipofectamine 2000 transfection reagent (Invitrogen) at 5 days in vitro (DIV5).

### 2.5 CMA activity assays

The pCDH‐KFERQ‐PA‐mCherry1‐N1 plasmid construction was performed following a previously described method.^16^ Photoconversion of cells grown on coverslips was carried out with a 405/20 nm LED array (Norlux) for 10 minutes using 50 mW/cm^2^ light intensity.

### 2.6 Real‐time quantitative polymerase chain reaction analysis

Total RNA was extracted from mouse brains using TRIzol reagent (Thermo Fisher Scientific). The RNA was reverse‐transcribed into cDNA using ReverTra Ace qPCR RT Master Mix (TOYOBO). The cDNA was analyzed by real‐time quantitative reverse transcription polymerase chain reaction (RT‐PCR; LightCycler 480 System) using the FastStart Universal SYBR Green Master (Roche). Target gene levels were normalized to those of β‐actin for comparisons. The primer sequences of studied genes are listed below:

*Il‐1β* Forward: 5′‐CAGGCAGGCAGTATCACTCATTG‐3′
*Il‐1β* Reverse: 5′‐GCTTTTTTGTTGTTCATCTCGGA‐3′
*Il‐6* Forward: 5′‐CAATGGCAATTCTGATTGTATG‐3′
*Il‐6* Reverse: 5′‐AGGACTCTGGCTTTGTCTTTC‐3′
*β‐actin* Forward: 5′‐AGCCATGTACGTAGCCATCCA‐3′
*β‐actin* Reverse: 5′‐TCTCCGGAGTCCATCACAATG‐3′
*Hspa8* Forward: 5′‐GTCACAGTGCCCGCTTACTT‐3′
*Hspa8* Reverse: 5′‐TCAAAAGTGCCACCTCCCAA‐3′

### 2.7 RNA‐seq analysis

Total RNA from the brain of 10‐month‐old male mice (four mice per genotype) was extracted using TRIzol reagent (Thermo Fisher Scientific) and then subjected to standard RNA‐seq conducted by Beijing Genomics Institute (BGI). Clean RNA‐seq data were analyzed for differentially expressed genes (DEGs) between different genotypes using BGI's Dr Tom platform (https://biosys.bgi.com) with default settings. Gene Ontology (GO) pathways of DEGs were also analyzed using the Dr Tom platform. The RNA‐seq data have been deposited into CNGB Sequence Archive (CNSA)^17^ of China National GeneBank DataBase (CNGBdb)^18^ with the accession number CNP0006258.

**RESEARCH IN CONTEXT**

1. **Systematic review**: We reviewed the literature from PubMed and Embase. Transmembrane protein 59 (TMEM59) is closely correlated with Alzheimer's disease (AD), the most common type of tauopathic disorder. However, whether and how TMEM59 regulates tau pathology in tauopathy remains unknown.
2. **Interpretation**: We first reported dramatically increased TMEM59 protein levels in the brains of AD patients and tau^P301S^ transgenic (PS19) mice. We then showed that TMEM59 haploinsufficiency attenuated cognitive deficits, neurodegeneration, synapse dysfunction, gliosis, neuroinflammation, and tau pathology in PS19 mice. Moreover, we found that TMEM59 interacted with heat‐shock cognate 71 kDa and lysosome‐associated membrane protein type 2A (LAMP2A) and TMEM59 deficiency markedly increased LAMP2A protein levels and promoted chaperone‐mediated autophagy (CMA) activity. These data indicate that TMEM59 may serve as a potential therapeutic target for tauopathy through regulating CMA activity.
3. **Future directions**: Detailed study on how TMEM59 regulates CMA may strengthen our understanding of the role of TMEM59 in tauopathy. Developing TMEM59‐reducing drugs using strategies such as anti‐sense oligonucleotides and targeted protein degradation may provide new candidates for tauopathy treatment.

### 2.8 Electrophysiology

Long‐term potentiation (LTP) was performed as previously described.^19^ Briefly, mice were anesthetized with isoflurane, and the brain was rapidly removed into an ice‐cold solution (64 mM NaCl, 2.5 mM KCl, 10 mM glucose, 1.25 mM NaH_2_PO_4_, 10 mM MgSO_4_, 26 mM NaHCO_3_, 120 mM sucrose, and 0.5 mM CaCl_2_). Slices were sectioned (400 µm) on a Leica VT1200S vibratome. The brain slices were incubated at 37°C for 1 hour and then recovered at room temperature for at least 1 hour before recording in an artificial cerebrospinal fluid (126 mM NaCl, 3.5 mM KCl, 1.25 mM NaH_2_PO_4_, 1.3 mM MgSO_4_, 2.5 mM CaCl_2_, 26 mM NaHCO_3_, and 10 mM glucose) with 95% O_2_/5% CO_2_. The field excitatory postsynaptic potentials (fEPSPs) were recorded in the Schaffer collateral pathway with a stimulated electrode placed in the CA3 stratum radiatum and a recording micropipette placed in the CA1 stratum radiatum. LTP was induced by two trains of high‐frequency stimulation (100 HZ, 1 second) with an interval of 30 seconds. Data were acquired with a Multiclamp 700B patch‐clamp amplifier (Molecular Devices) and analyzed using pClamp software (Molecular Devices, version 10.6).

### 2.9 Western blotting

Mouse brain tissues were lysed in radio immunoprecipitation assay lysis buffer (25 mM Tris–HCl [pH 7.6], 150 mM NaCl, 0.1% sodium dodecyl sulfate, 1% sodium deoxycholate, and 1% Nonidet P‐40, supplemented with protease inhibitors and phosphatase inhibitors). Cultured cells were lysed in TNEN lysis buffer (20 mM Tris‐HCl, pH 8.0, 100 mM NaCl, 1 mM ethylenediaminetetraacetic acid, and 0.5% NP‐40, supplemented with protease and phosphatase inhibitors). Protein concentrations were measured by a BCA Protein Assay Kit (Thermo Fisher Scientific). Ten to twenty micrograms of protein lysates were subjected to sodium dodecyl sulfate polyacrylamide gel electrophoresis and western blotting. The blots were immunoblotted with primary antibodies, including: anti‐β‐actin (CST, 8457S, 1:2000), anti‐ATF6 (Abcam, ab227830, 1:1000), anti‐BiP (CST, C50B12, 1:1000), anti‐CDK5 (Santa Cruz, sc‐173, 1:1000), anti‐Flag (Proteintech, 20543‐1‐AP, 1:1000), anti‐GAPDH (Abways, AB0038, 1:5000), anti‐GFAP (Cell Signaling Technology, 3670S, 1:1000), anti‐GFP (Proteintech, 50430‐2‐AP, 1:1000), anti‐GluA2 (CST, 13607S, 1:1000), anti‐GSK3β (Proteintech, 51065‐1‐AP, 1:1000), anti‐HA (Proteintech, S1064‐2‐AP, 1:1000), anti‐HSC70 (Proteintech, 10654‐1‐AP, 1:1000), anti‐Iba1 (Wako, 016‐20001, 1:500), anti‐LAMP2A (Abcam, ab18528, 1:1000), anti‐LC3B (CST, 3868, 1:500), anti‐myc (CST, 2276S, 1:1000), anti‐p35/25 (CST, 2680, 1:1000), anti‐PERK (CST, D11A8, 1:1000), anti‐PP1α (Thermo, 43‐8100, 1:1000), anti‐PP2A‐α (ABclonal, A6702, 1:1000), anti‐PP2B‐Aα (Santa Cruz, sc‐17808, 1:1000), anti‐pPERK (CST, 16F8, 1:1000), anti‐pS202/T205 tau (AT8, Invitrogen, MN1020, 1:500), anti‐pS212 tau (Biosource, 44‐740G, 1:1000), anti‐pS396 tau (Invitrogen, 44752G, 1:1000), anti‐pT216‐GSK3β (Proteintech, 51065‐1‐AP, 1:1000), anti‐total tau (Tau5, Invitrogen, AHB0042, 1:1000), anti‐TMEM59 (ABclonal, WG‐03224D, 1:1000), and anti‐XBP1 (Abcam, ab220783, 1:1000). Secondary antibodies used were horseradish peroxidase‐conjugated second antibodies (Thermo Fisher Scientific, 31430 or 31460; 1:5000). The immunoreactive bands were quantified using the National Institutes of Health's ImageJ software.

### 2.10 Immunostaining

Immunostaining was carried out as previously described.^19^ Primary antibodies used included: anti‐CD68 (Biolegend, 137001, 1:300), anti‐GFAP (Proteintech, 16825‐1‐AP, 1:200), anti‐Iba1 (Wako, 016‐20001, 1:200), anti‐myc (CST, 2276S, 1:400), anti‐NeuN (CST, 94403S, 1:200), anti‐pS202/T205 tau (AT8, Invitrogen, MN1020, 1:200), anti‐PSD95 (Millipore, MAB1596, 1:200), and anti‐synapsin 1 (Proteintech, 20258‐1‐AP, 1:200). Secondary antibodies used were from Thermo Fisher Scientific (A‐11008, A‐21202, A11012, and A‐11005, 1:400). DAPI was from Sigma‐Aldrich (D95542; 1 µg/mL). Images were captured with the A1R (Nikon) or FV1000MPE‐B (Olympus) confocal microscope. For NeuN, phospho‐Tau, and CD68% area calculation, images were processed using ImageJ software and with the following operations: select image type as “8‐bit,” adjust the threshold, and use the “analyze particles” function to calculate %area. For microglial morphology analysis, 3D reconstruction images were reconstructed and analyzed using the “filaments” and “surface” functions in the Imaris 10.0.0 software as described previously.^20^ For microglial arborization quantification, Sholl analysis was performed using the Imaris software as previously described.^21^, ^22^, ^23^

### 2.11 Golgi staining

Mouse brains were rapidly dissected and sliced (150 µm thickness) for Golgi staining using the FD Rapid Golgistain Kit (FD Neuro Technologies) and following the manufacturer's instructions. CA1 neurons were captured with the Olympus FV1000 MPE‐B confocal microscope, and the spine numbers were measured using Image J software.

### 2.12 Bimolecular fluorescence complementation assay

Full‐length human HSC70 cDNA was cloned into the pBiFC‐VN173 vector containing the N‐terminal part of Venus, and full‐length LAMP2A cDNA was cloned into the pBiFC‐VN155 vector containing the C‐terminal part of Venus. HEK293T cells were co‐transfected with HSC70‐VN173 and LAMP2A‐VC155. Green fluorescence representing the HSC70–LAMP2A interaction was observed under a confocal microscope.

### 2.13 Protein kinase activity and phosphatase activity test

Mouse brain tissues were lysed in cell lysis buffer (Beyotime, P0013J). Equal protein amounts of lysates were measured for protein kinase activity and phosphatase activity using a Kinase‐Lumi Luminescent Kinase Assay Kit (Beyotime, S0150S) and an Alkaline Phosphatase Assay Kit (Beyotime, S0321S), respectively, following the manufacturer's instructions.

### 2.14 Behavioral tests

#### 2.14.1 Open field test

Mice were placed in the center of an open field (40 cm × 40 cm × 40 cm) for 10 minutes. The total distance traveled and the time spent in the center were scored by Smart Video Tracking Software 3.0.

#### 2.14.2 Y‐maze test

Mice were placed in the center of a Y‐shaped maze with three arms at 120°C (30 cm [L] × 6 cm [W] × 15 cm [H]). Each mouse was allowed to freely explore the maze for 5 minutes. The percentage of alternation was calculated using Smart Video Tracking Software 3.0.

#### 2.14.3 Novel object recognition test

This test contains habituation, training, and testing phases on separate days. Mice were first allowed to habituate an open field for 10 minutes (40 cm × 40 cm × 40 cm) on day 1. During training on day 2, mice were allowed to explore two identical objects (A and B) for 10 minutes. In the test phase on day 3, object B was replaced with a novel object C, and mice were allowed to explore the two objects (A and C) for 10 minutes. The time spent exploring each object was measured for comparison. The discrimination index (DI) was calculated as DI = T_C_/ (T_A_ + T_C_). T_C_ and T_A_ are time spent on object A and object C in the test phase, respectively.

#### 2.14.4 Morris water maze test

This test was performed in a circular pool (120 cm in diameter) filled with opaque water held at 22 ± 2°C. A hidden platform was submerged 1 cm below the surface. Four different shapes as cues were affixed to the tank. Mice were trained for 7 consecutive days with two training trials per day. Each mouse was allowed to search the hidden platform for 60 seconds. If the mouse lacked the ability to find the platform within 60 seconds, it was guided to the platform and allowed to stay there for 10 seconds. During the test on day 8, the hidden platform was removed, and mice were allowed to perform for 60 seconds. The time spent in each quadrant and the number of crossings of the platform region were recorded.

### 2.15 Statistics analyses

Statistical analyses, including Student *t* test, one‐way analysis of variance (ANOVA) with Tukey post hoc analysis, and two‐way ANOVA with Tukey post hoc analysis, were performed using GraphPad Prism 8. A detailed method for each statistical comparison is provided in the corresponding figure legend. All data represent mean ± standard error of the mean. *P* < 0.05 is considered statistically significant.

## 3 RESULTS

### 3.1 TMEM59 expression is increased in the brains of AD patients and PS19 mice at pathological stages

**FIGURE 1:**
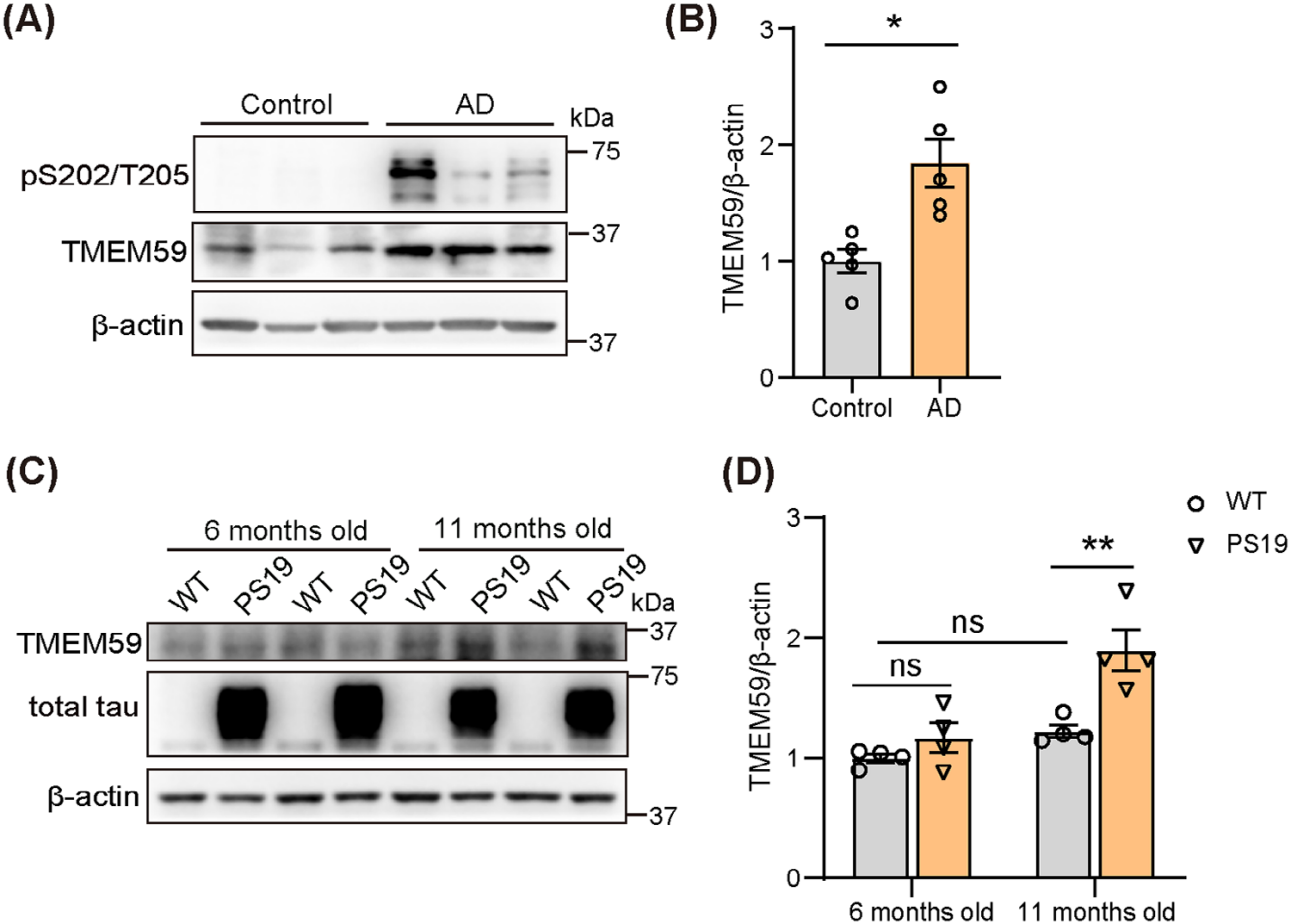
TMEM59 expression is increased in the brains of AD patients and PS19 mice at pathological stages. Western blotting (A) and quantitative analysis (B) of TMEM59 protein levels in the brains of controls and AD patients. Tau phosphorylated at sites Ser202/Thr205 was used for AD confirmation. *n* = 5 samples per group. Western blotting (C) and quantitative analysis (D) of TMEM59 protein levels in the hippocampi of 6‐month‐old and 11‐month‐old PS19 tauopathy mouse model (PS19 mice). *n* = 4 mice per group. Data represent mean ± standard error of the mean. *P* values were determined by two‐way analysis of variance with Tukey post hoc analysis. ns, not significant; *P < 0.05; **P < 0.01. AD, Alzheimer's disease; PS19, tau^P301S^ transgenic; TMEM59, transmembrane protein 59; WT, wild type.

We examined TMEM59 protein levels in *post mortem* brain samples from individuals with AD and age‐matched controls and found that TMEM59 protein levels were increased in AD patients (Figure 1A,B). We also examined TMEM59 protein levels in the hippocampus of PS19 mice at different ages. The results showed that although TMEM59 protein levels were comparable between 6‐month‐old and 11‐month‐old wild‐type (WT) mice and between PS19 mice and WT controls at 6 months of age, they significantly increased in PS19 mice at 11 months of age when disease‐like phenotypes become dramatic^24^, ^25^ (Figure 1C,D).

### 3.2 TMEM59 haploinsufficiency attenuates memory deficits in PS19 mice

**FIGURE 2:**
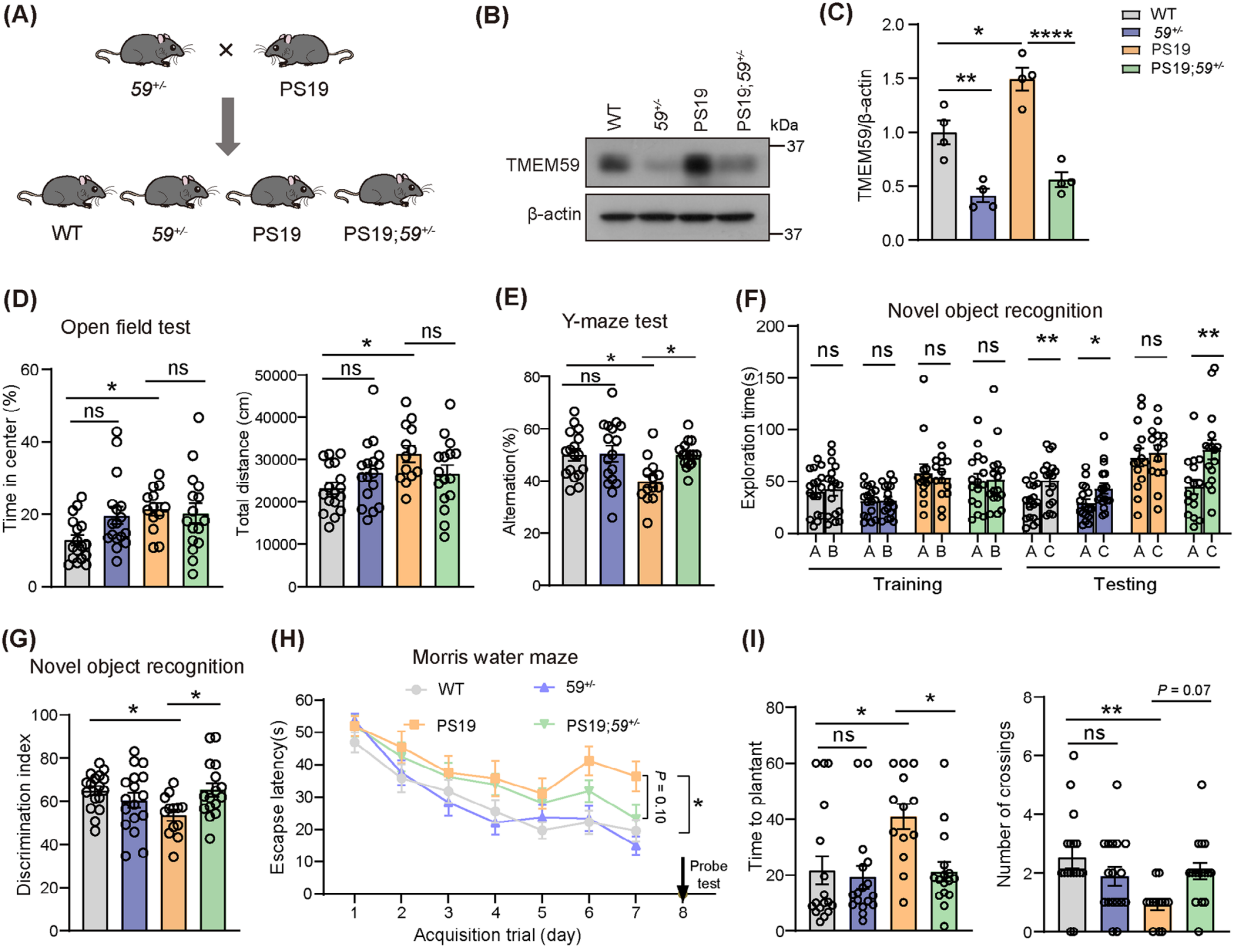
*Tmem59* haploinsufficiency attenuates memory deficits in PS19 mice. A, Schematic diagram for generating PS19 mice with *Tmem59* haploinsufficiency. Western blotting (B) and quantitative analysis (C) of TMEM59 protein levels in 9.5‐ to 10‐month‐old mouse brain lysates. *n* = 4 mice per group. D–I, 9.5‐ to 10‐month‐old mice were subjected to behavioral analyses of total travel distance and duration in the center in the open field test (D), of the spontaneous alternation percentage in the Y maze test (E), of the exploration time (F) and the discrimination index (G) in the novel object recognition test, and of the escape latency during a 7‐day training phase (H) and the time spent reaching the platform place and the number of platform place crossings during the testing phase on day 8 (I) in the Morris water maze test. WT *n* = 17 mice, *59^+/−^* (*Tmem59^+/−^*) *n* = 17 mice, PS19 *n *= 13 mice, and PS19;*59^+/−^* (PS19;*Tmem59^±^*) *n *= 16 mice. Data represent mean ± standard error of the mean. *P* values were determined by one‐way ANOVA with Tukey post hoc analysis in (C, D, E, G, and I), unpaired *t* test in (F), and two‐way ANOVA with Tukey post hoc analysis in (H). ns, not significant; *P < 0.05; **P < 0.01; ****P < 0.0001. AD, Alzheimer's disease; ANOVA, analysis of variance; PS19, tau^P301S^ transgenic; TMEM59, transmembrane protein 59; WT, wild type.

Because TMEM59 expression is increased in the brain of AD patients and tauopathy model mice, we next studied whether TMEM59 reduction can alter memory and synaptic plasticity in PS19 mice. We generated WT (*Tmem59^+/+^*), *Tmem59^±^* (*59^+/−^*), PS19, and PS19;*Tmem59^±^* (PS19;*59^+/−^*) mice by crossing 59^+/−^ mice with PS19 mice (Figure 2A). TMEM59 protein levels were confirmed to significantly decrease in *59^+/−^* and PS19;*59^+/−^* mice compared to WT and PS19 mice, respectively (Figure 2B,C). TMEM59 protein levels were also found to increase in 9.5‐ to 10‐month‐old PS19 mice compared to WT controls (Figure 2B,C).

These mice were subjected to various behavioral tests at 9.5 to 10 months of age. In the open field test, PS19 mice had more total travel distance and spent more time in the center than WT mice, whereas TMEM59 haploinsufficiency had no effect on altering mouse locomotor activity in both WT and PS19 mice (Figure 2D). In the Y maze test, PS19 mice had decreased spontaneous alternation percentage compared to WT controls (Figure 2E). In the novel object recognition test, although PS19 mice explored two identical objects (A and B) similarly during the training phase, they failed to recognize a novel object (C) and had a reduced discrimination index compared to WT controls during the testing phase (Figure 2F,G). In the Morris water maze test, PS19 mice showed more escape latency than WT controls during the training phase (Figure 2H). They spent more time reaching the platform place for the first time and had fewer numbers of platform place crossings than WT controls during the testing phase (Figure 2I). Therefore, PS19 mice develop learning and memory deficits at 9.5 to 10 months of age. Notably, compared to PS19 mice, PS19;*59^+/−^* mice exhibited increased spontaneous alternation percentage in the Y maze test (Figure 2E), elevated discrimination index to distinguish the novel and the familiar object in the novel objection recognition test (Figure 2F,G), and less time to reach the platform place for the first time during the testing phase of the Morris water maze test (Figure 2I), while *59^+/−^* mice showed no changes in learning and memory compared to WT mice (Figure 2E–I). Together, these results indicate that TMEM59 haploinsufficiency can ameliorate memory deficits in PS19 mice.

### 3.3 TMEM59 haploinsufficiency attenuates neurodegeneration and synapse dysfunction in PS19 mice

**FIGURE 3:**
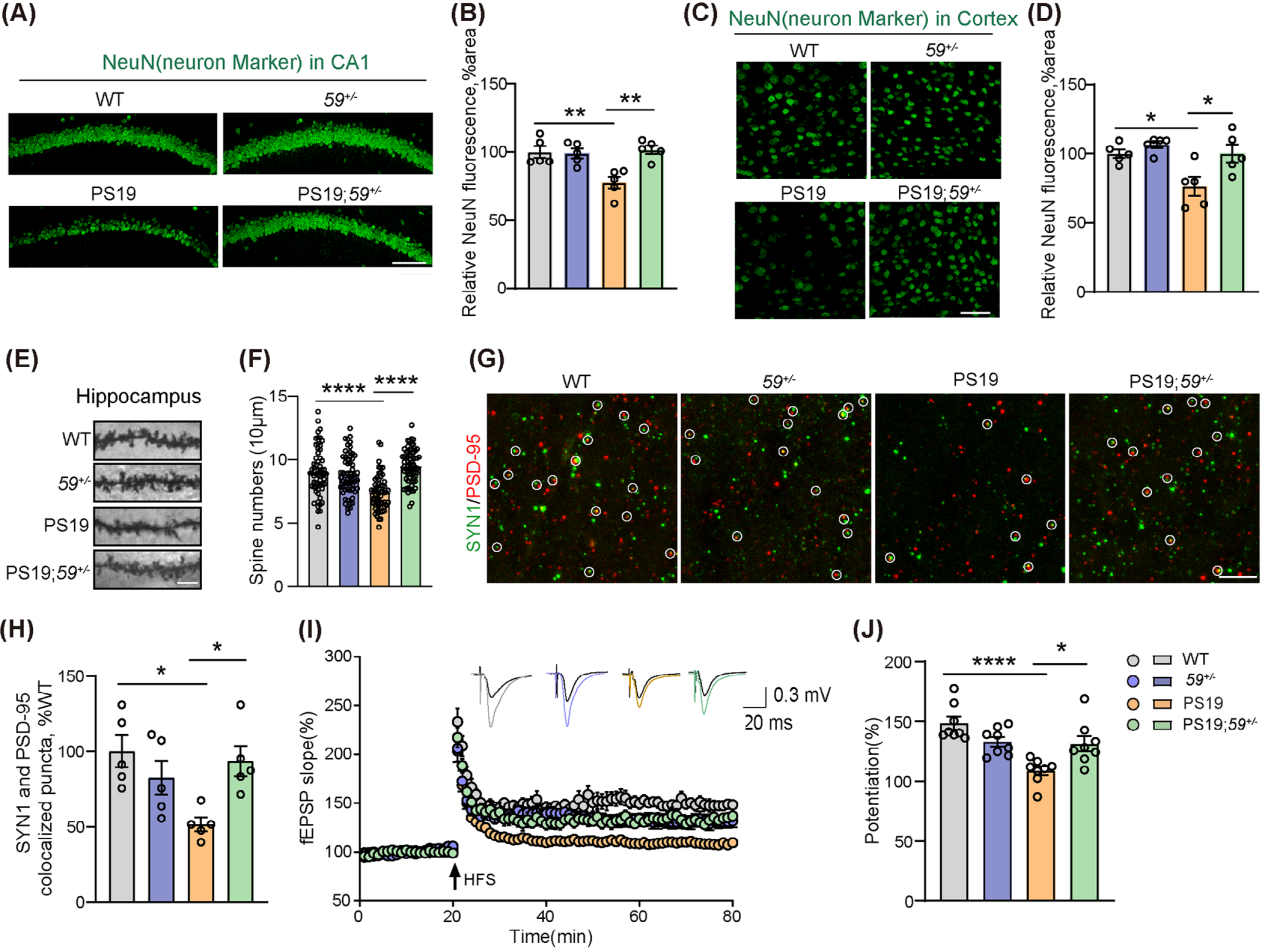
*Tmem59* haploinsufficiency attenuates neurodegeneration and synaptic plasticity deficits in PS19 mice. A–D, Neurons in the hippocampal CA1 (A, B) and cortical (C, D) regions of 10‐month‐old mice were stained with the neuronal marker NeuN (in green, A, C) for comparisons (B, D). *n* = 5 mice per group. Scale bars, 100 µm. Golgi staining (E) and quantification of dendritic spines (F) in hippocampal CA1 area. *n* = 59‐64 dendrites from four mice per group. Scale bar, 5 µm. Representative high‐magnification confocal images of SYN1 (in green) and PSD‐95 (in red) co‐immunostaining in CA1 (G) and comparisons of SYN1/PSD‐95 colocalized puncta (H). *n* = 5 mice per group. Scale bar, 5 µm. I and J, Hippocampal CA1 long‐term potentiation was recorded in 10‐month‐old mice (I). Mean fEPSP potentiation was quantified at 50 to 60 minutes after high‐frequency stimulation (HFS) for comparisons (J). WT *n* = 8 slices from five mice, *59^+/−^ n *= 8 slices from four mice, PS19 *n *= 8 slices from four mice, and PS19;*59^+/−^ n *= 8 slices from four mice. Data represent mean ± standard error of the mean. *P* values were determined by one‐way analysis of variance with Tukey post hoc analysis. *P < 0.05; **P < 0.01; ****P < 0.0001. AD, Alzheimer's disease; fEPSP, field excitatory postsynaptic potential; PS19, tau^P301S^ transgenic; PSD‐95, postsynaptic density protein 95; SYN1, synapsin 1; TMEM59, transmembrane protein 59; WT, wild type.

To further study the effects of TMEM59 haploinsufficiency on neurodegeneration, we compared NeuN‐positive cells in different mice. As expected, PS19 mice had reduced NeuN immunoreactivity in both hippocampal CA1 and cortical regions, whereas TMEM59 haploinsufficiency reversed such reductions in PS19 mice (Figure 3A–D). Golgi staining also revealed that the hippocampal neurons of PS19 mice had decreased dendritic spine density, which was reversed upon TMEM59 haploinsufficiency (Figure 3E,F). When the presynaptic marker synapsin 1 (SYN1) and the postsynaptic marker postsynaptic density protein 95 (PSD‐95) were co‐immunostained, we also observed that PS19 mice had reduced colocalization of the two, whereas TMEM59 haploinsufficiency reversed such a reduction (Figure 3G,H).

To further determine the effect of TMEM59 deficiency on synaptic plasticity, we measured LTP in hippocampal Schaffer collaterals and found that although TMEM59 haploinsufficiency had no effect on LTP at basal levels, it rescued impaired LTP in PS19 mice (Figure 3I,J). These results suggest that TMEM59 haploinsufficiency rescues neurodegeneration and synaptic plasticity deficits in PS19 mice. One previous study found that complete deletion of TMEM59 could reduce Aβ pathologies and rescue reduced levels of GluA2, a component of the α‐amino‐3‐hydroxy‐5‐methyl‐4‐isoxazolepropionic acid (AMPA) receptor in the APP/PS1 AD model mice that carry the APP Swedish mutation and the PS1 ΔE9 mutation.^13^ However, we found that although GluA2 levels decreased in PS19 mice, TMEM59 haploinsufficiency did not reverse the GluA2 reduction (Figure S1A,B in supporting information). Whether complete deletion of TMEM59 can rescue GluA2 reduction in PS19 mice deserves further scrutiny.

### 3.4 TMEM59 haploinsufficiency suppresses gliosis and neuroinflammation in PS19 mice

**FIGURE 4:**
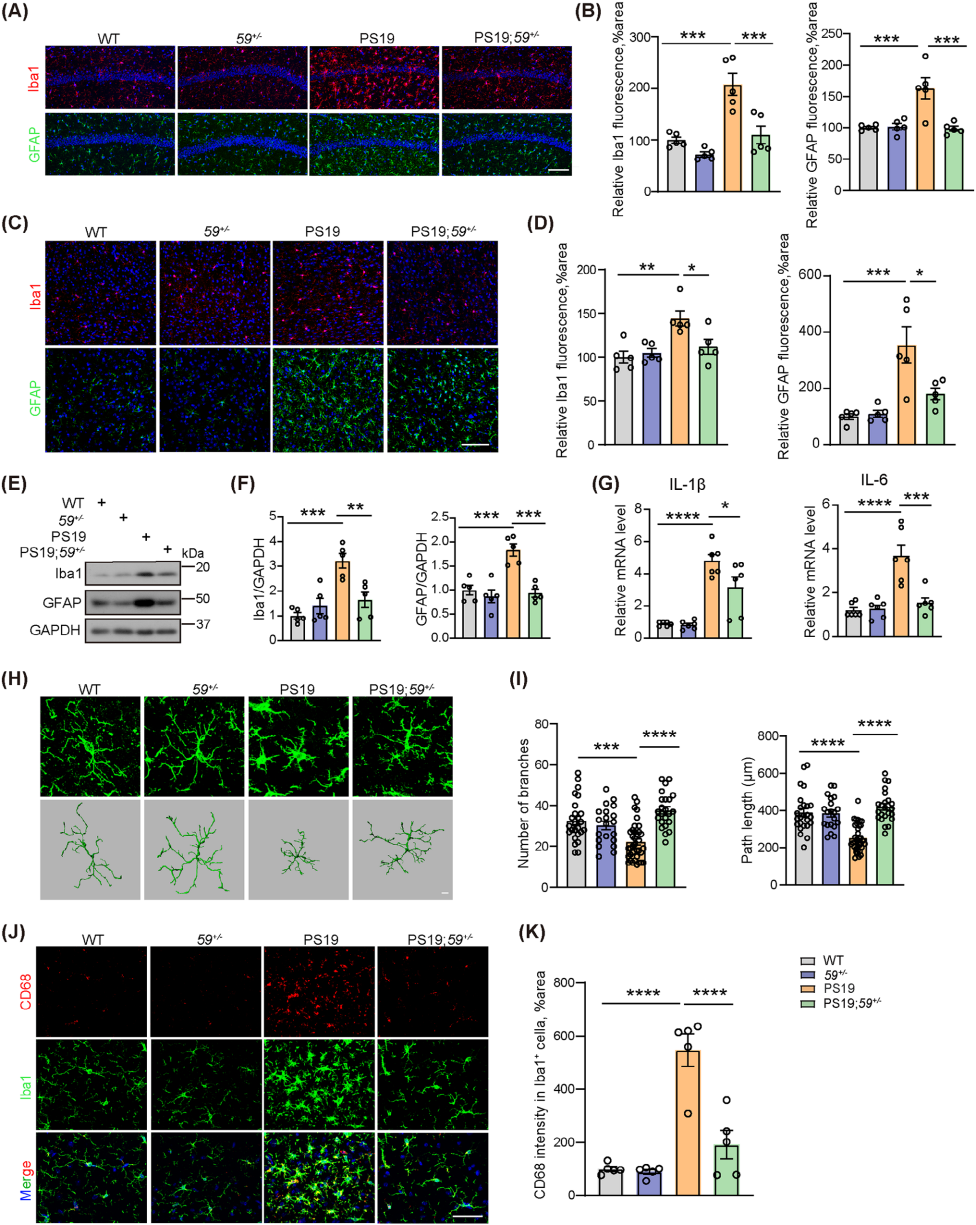
*Tmem59* haploinsufficiency suppresses reactive gliosis and neuroinflammation in PS19 mice. Representative immunostaining of Iba1^+^ microglia (in red) and GFAP^+^ astrocytes (in green) in the hippocampal CA1 (A) and cortical (C) regions of 10‐month‐old mice and their quantitative comparisons (B, D). The nuclei were stained by DAPI (in blue, A). *n* = 5 mice per group. Scale bars, 100 µm. Western blotting (E) and quantitative comparisons (F) of Iba1 and GFAP protein levels in 9.5‐ to 10‐month‐old mouse brain lysates. *n* = 5 mice per group. G, Quantitative polymerase chain reaction analyses of IL‐1β and IL‐6 mRNA levels in the mouse brain. *n* = 6 mice per group. Representative 3D reconstruction of Iba1‐immunostained microglia (in green) in the mouse brain (H) and quantitative analyses of microglial branch numbers and path lengths (I). Scale bar, 5 µm. *n* = 20 to 36 cells from five mice per group. Representative co‐immunostaining of Iba1 (in green) and CD68 (in red) in the hippocampal CA1 (J) and quantitative comparisons of CD68 in Iba1^+^ microglia (K). Scale bar, 50 µm. *n* = 5 mice per group. Data represent mean ± standard error of the mean. *P* values were determined by one‐way analysis of variance with Tukey post hoc analysis. *P < 0.05; **P < 0.01; ***P < 0.001; ****P < 0.0001. AD, Alzheimer's disease; CD68, cluster of differentiation 68; GAPDH, glyceraldehyde 3‐phosphate dehydrogenase; GFAP, glial fibrillary acidic protein; Iba1, ionized calcium‐binding adaptor molecule 1; IL, interleukin; PS19, tau^P301S^ transgenic; TMEM59, transmembrane protein 59; WT, wild type.

Next, we assessed the impact of TMEM59 haploinsufficiency on microgliosis and astrogliosis by immunostaining ionized calcium‐binding adaptor molecule 1 (Iba1) and glial fibrillary acidic protein (GFAP), respectively. We found that both Iba1 and GFAP immunoreactivities were significantly increased in the hippocampal CA1 and cortical regions of PS19 mice compared to WT controls, whereas TMEM59 halploinsufficiency markedly reversed such increases (Figure 4A–D). Consistently, western blot analysis showed that PS19 mouse brain lysates had significantly increased Iba1 and GFAP protein levels, which were reduced upon TMEM59 haploinsufficiency (Figure 4E,F). Moreover, we studied and found that the mRNA levels of two proinflammatory factors, interleukin (IL)‐1β and IL‐6, were dramatically increased in PS19 mice, and such increases were reduced upon TMEM59 haploinsufficiency (Figure 4G). When 3D reconstruction of microglia was carried out, we found that microglia in PS19 mice exhibited decreased branch numbers and path lengths, which were reversed upon TMEM59 haploinsufficiency (Figure 4H,I). To further characterize microglia activation states, we performed cluster of differentiation 68 (CD68) and Iba1 double immunostaining to identify CD68‐positive microglia (Figure 4J). Quantification analysis revealed that TMEM59 haploinsufficiency significantly reduced CD68 immunoreactivity in Iba1‐positive microglia in PS19 mice (Figure 4K). These results demonstrate that TMEM59 deficiency can attenuate microgliosis, astrogliosis, and neuroinflammation in PS19 mice.

### 3.5 TMEM59 deficiency reduces tau phosphorylation in PS19 mice and accelerates the degradation of phosphorylated tau

**FIGURE 5:**
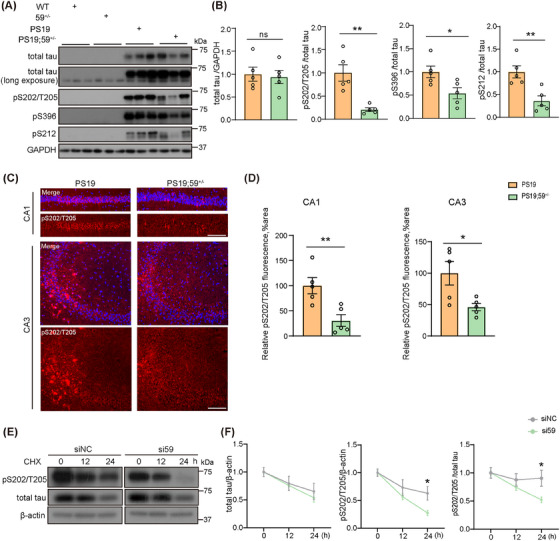
*Tmem59* deficiency reduces tau pathology. Western blotting (A) and quantitative comparisons (B) of total tau and tau phosphorylated at sites Ser202/Thr205 (pS202/T205), Ser396 (pS396), and Ser212 (pS212) in the hippocampal lysates of 10‐month‐old PS19 and PS19;*59^+/−^* mice. *n* = 5 mice per group. Representative immunostaining images of pS202/T205 (in red, C) and their quantitative comparisons (D) in mouse hippocampal CA1 and CA3 regions. The nuclei were stained by DAPI (in blue, C). *n* = 5 mice per group. Scale bars, 100 µm. E, HEK293T cells expressing tau were transfected with TMEM59 targeting siRNA (si59) or control siRNA (siNC) and then treated with cycloheximide (CHX) for indicated time points. Protein lysates were subjected to western blotting of indicated proteins. F, Total tau levels were normalized to β‐actin levels for comparison. pS202/T205 tau levels were normalized to β‐actin levels and total tau levels, respectively, for comparison. *n* = 6 per group. Data represent mean ± standard error of the mean. *P* values were determined by unpaired *t* test in (B and D) and two‐way analysis of variance with Tukey post hoc analysis in (F). ns, not significant; *P < 0.05; **P < 0.01. AD, Alzheimer's disease; GAPDH, glyceraldehyde 3‐phosphate dehydrogenase; PS19, tau^P301S^ transgenic; TMEM59, transmembrane protein 59; WT, wild type.

Tau hyperphosphorylation is a central pathology in tauopathy. We found that TMEM59 haploinsufficiency had no effect on total tau levels but significantly decreased tau phosphorylation at sites S202/T205, S396, and S212 in PS19 mouse brain lysates (Figure 5A,B). Immunostaining results confirmed that tau S202/T205 phosphorylation in the CA1 and CA3 regions of PS19 mice was significantly reduced upon TMEM59 haploinsufficiency (Figure 5C,D). In HEK293T cells expressing the tau P301L mutation, we also found that TMEM59 downregulation reduced tau phosphorylation at sites S202/T205 (Figure S2A,B in supporting information).

To explore the mechanism underlying TMEM59‐mediated tau phosphorylation, we first assayed several protein kinases and phosphatases known to phosphorylate/dephosphorylate tau in brain samples of different mice. However, we observed no change in the protein levels of studied protein kinases (GSK3β and its phosphorylated form, and CDK5 and its cofactors p25/p35) and phosphatases (PP1, PP2A, and PP2B; Figure S3A,B in supporting information). Moreover, we measured general kinase and phosphatase activities in brain samples of different mice and found no changes (Figure S3C,D). These results imply that TMEM59 haploinsufficiency has no effect on the activities of tau kinases and phosphatases.

We then studied whether TMEM59 affects tau degradation. In HEK293T cells expressing the tau P301L mutation, we found that TMEM59 downregulation had no effect on total tau degradation but markedly accelerated the degradation of phosphorylated tau (S202/T205 sites; Figure 5E,F), implying that TMEM59 haploinsufficiency reduces tau phosphorylation through promoting phosphorylated tau degradation.

### 3.6 TMEM59 deficiency activates CMA via increasing LAMP2A protein levels

We carried out RNA‐seq for the brain tissues of different mice at 10 months of age. Between the 1256 DEGs identified in the PS19 versus WT group (Table S2 in supporting information) and the 573 DEGs identified in the PS19;*59^+/−^* versus PS19 group (Table S3 in supporting information), there were 129 overlapping DEGs (Figure S4A and Table S4 in supporting information). A total of 32 of the 129 overlapping DEGs were consistently increased or decreased in both the PS19 versus WT and the PS19;*59^+/−^* versus PS19 groups (Figure S4B and Table S5 in supporting information) while the other 97 DEGs had different change directions (increased in one and decreased in the other one, or vice versa) in the two groups (Figure S4B,C and Table S6 in supporting information).

**FIGURE 6:**
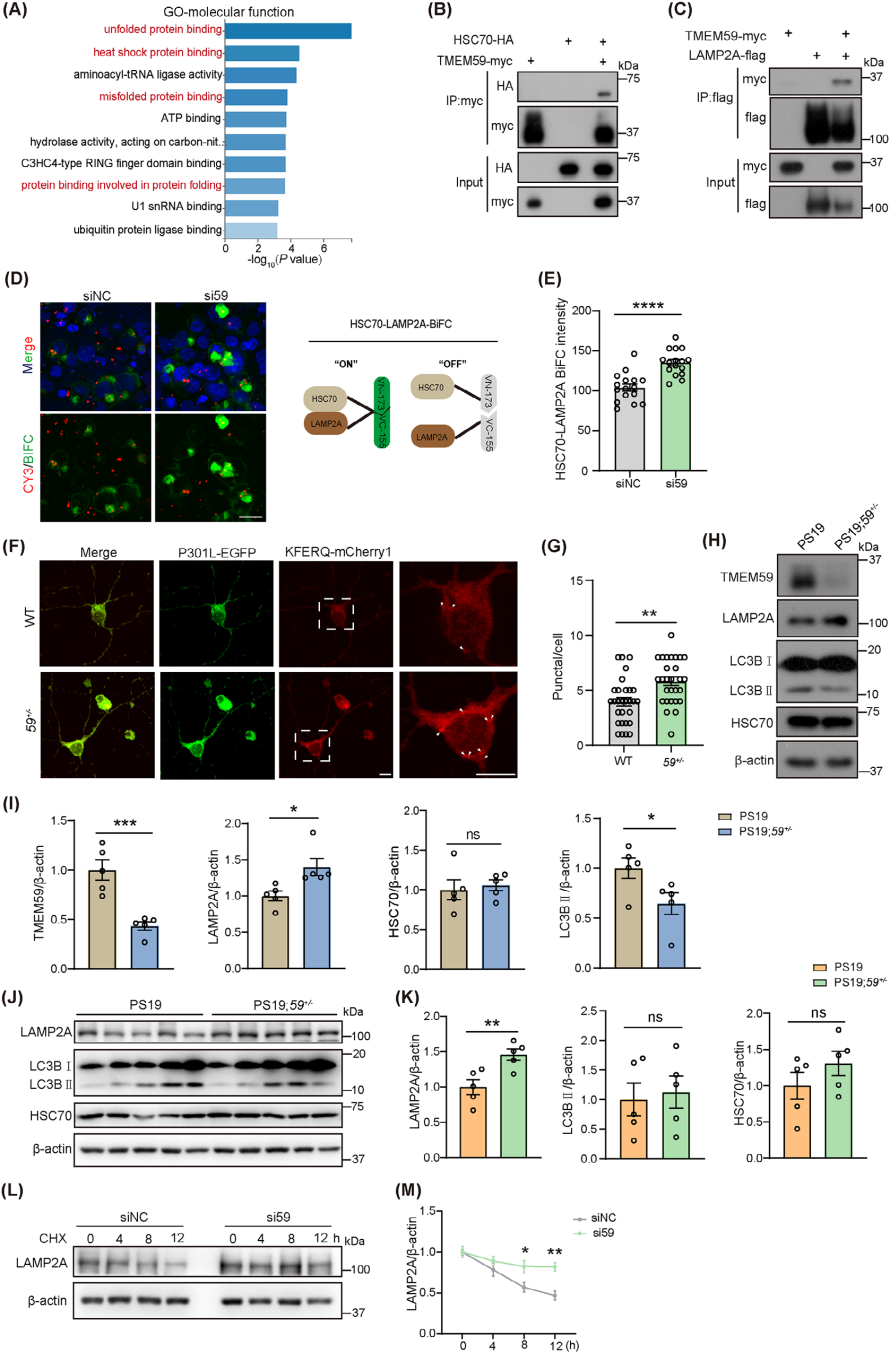
*Tmem59* deficiency activates CMA and increases LAMP2A protein levels. A, The 97 differentially expressed genes with different change directions between the PS19 versus WT group and the PS19;*59^+/−^* versus PS19 group were subjected to Gene Ontology (GO) analysis for molecular functions. TMEM59‐myc was co‐transfected with HSC70‐HA (B) or LAMP2A‐flag (C) in HEK293T cells. Equal amounts of protein lysates were immunoprecipitated with an anti‐myc antibody and immunoblotted with anti‐myc and anti‐HA antibodies (B), or immunoprecipitated with an anti‐flag antibody and immunoblotted with anti‐myc and anti‐flag antibodies (C). D, HEK293T cells were first co‐transfected with HSC70‐VN173 and LAMP2A‐VC155 and then transfected with fluorescence‐labeled TMEM59 targeting siRNA (si59) or control siRNA (siNC). Cells were stained with DAPI (in blue) and observed under a confocal microscope. The scheme for the HSC70–LAMP2A bimolecular fluorescence complementation assay is also shown. The green color represents the HSC70–LAMP2A interaction. The red color indicates si59 or siNC. Scale bar, 20 µm. E, Quantitative comparison of green intensities in cells containing red color in (D). *n* = 16 cells per group. F and G, Primary neurons from WT and *59^+/−^* mice at E16.5 were co‐transfected with KFERQ‐PA‐mCherry1 (in red) and P301L‐EGFP (in green) at DIV5. Cells were photoconverted at DIV6. At DIV8, cells were observed under a confocal microscope (F). Red puncta in GFP‐expressing neurons were quantified for comparisons (G). Scale bar, 10 µm. *n* = 28–29 cells from four mice per group. Western blotting of TMEM59, LAMP2A, HSC70, and LC3B in primary neurons derived from PS19 and PS19;*59* ^+/−^ mice (H) and their quantitative comparisons (I). *n* = 5 mice per group. Western blotting of LAMP2A, LC3B, and HSC70 in mouse brain lysates (J) and their quantitative comparisons (K). *n* = 5 mice per group. L, HEK293T cells expressing tau were transfected with si59 or siNC and then treated with cycloheximide (CHX) for indicated time points. Protein lysates were subjected to western blotting of indicated proteins. M, LAMP2A levels were normalized to β‐actin levels for comparison. *n* = 5 per group. Data represent mean ± standard error of the mean. *P* values were determined by unpaired *t* test in (E, G, I, and K) and two‐way analysis of variance with Tukey post hoc analysis in (M). ns: not significant; *P < 0.05; **P < 0.01; ***P < 0.001; ****P < 0.0001. AD, Alzheimer's disease; CMA, chaperone‐mediated autophagy; GFP, green fluorescent protein; HSC70, heat‐shock cognate 71 kDa; LAMP2A, lysosome‐associated membrane protein type 2A; LC3B, microtubule‐associated protein 1 light‐chain 3B; PS19, tau^P301S^ transgenic; TMEM59, transmembrane protein 59; WT, wild type.

Because genes whose expression is altered in the PS19 versus WT group and reversed in the PS19;*59^+/−^* versus PS19 group may play important roles in TMEM59 haploinsufficiency‐exerted protection in PS19 mice, we performed GO enrichment analysis of the 97 DEGs with different change directions in the two groups. GO molecular function analysis revealed that many of these DEGs were enriched in “unfolded protein binding,” “heat shock protein binding,” “misfolded protein binding,” and “protein binding involved in protein folding” pathways (Figure 6A). Gene set enrichment analysis (GSEA) revealed that DEGs in both PS19 versus WT and PS19;*59^+/−^* versus PS19 groups were correlated with a misfolded protein pathway (Figure S4E). However, we found that TMEM59 haploinsufficiency had no effect on altering the levels of BiP, ATF6, PERK, phosphorylated PERK, spliced XBP1, and unspliced XBP1 that are related to the unfolded protein response (UPR, Figure S5A,B), implying that TMEM59 haploinsufficiency does not affect the UPR pathway.

On the other hand, to identify TMEM59‐binding proteins, we transfected HEK293T cells with a TMEM59‐myc plasmid or a control myc plasmid and then carried out immunoprecipitation (IP) using an anti‐myc antibody. Some proteins with molecular weights of ≈ 70 kDa were found to be specifically immunoprecipitated by the anti‐myc antibody but not by immunoglobulin G in cells expressing TMEM59 and not in control cells (Figure S6A in supporting information). Mass spectrometry analysis of these proteins identified multiple heat shock proteins, including HSC70, which plays an important role in CMA (Table S7 in supporting information). Interestingly, the mRNA levels of the HSC70 encoding gene *Hspa8* were found to significantly increase in the brain of PS19 mice, and such increases were reduced upon TMEM59 haploinsufficiency (Figure S4C,D). During CMA, substrate proteins are recruited to the lysosomes by HSC70, which binds to LAMP2A on the lysosomal membrane to facilitate substrate translocation into the lysosomal lumen for degradation.^26^ Because CMA has been reported to mediate the degradation of pathogenic proteins, including tau,^5^, ^6^, ^7^ we studied and confirmed the interaction between HSC70 and TMEM59 using co‐immunoprecipitation assays (Figure 6B). Moreover, we found that TMEM59 also interacted with LAMP2A (Figure 6C). However, although we confirmed previous studies showing that HSC70 binds tau,^27^ we found that TMEM59 had no interaction with tau (Figure S6B). We subsequently studied whether TMEM59 affects the binding between HSC70 and LAMP2A by first generating a bimolecular fluorescence complementation assay for HSC70 and LAMP2A, in which the HSC70–LAMP2A interaction is indicated by green fluorescence (Figure 6D and Figure S6C). We found that in this assay, TMEM59 downregulation and overexpression markedly increased and decreased the fluorescence signal, respectively (Figure 6D,E and S6C,D), suggesting that TMEM59 downregulation promotes the HSC70–LAMP2A interaction, whereas TMME59 overexpression has the opposite effect. Co‐immunoprecipitation assays also confirmed that the HSC70–LAMP2A interaction was decreased upon TMEM59 overexpression (Figure S6E–H).

To further determine whether TMEM59 regulates the CMA activity, we monitored CMA in cultured cells using photoswitchable mCherry1 fluorescent protein tagged with the CMA‐targeting motif (KFERQ‐PS‐mCherry1), an assay previously established for studying CMA.^28^ Upon CMA activation by serum deprivation, KFERQ‐PS‐mCherry1 relocalized from the cytosol to the lysosomal surface, thereby showing a punctate fluorescent pattern (Figure S6I,J). Using this assay, we found that the CMA activity was elevated in TMEM59 haploinsufficient primary neurons compared to WT primary neurons (Figure 6F,G). Meanwhile, TMEM59 overexpression decreased the CMA activity in HEK293T cells with or without serum deprivation (Figure S6I,J).

We next measured HSC70 and LAMP2A protein levels in primary neurons derived from PS19 and PS19;*59^+/−^* mice and found that TMEM59 haploinsufficiency significantly increased protein levels of LAMP2A and decreased protein levels of LC3B II, a marker for macroautophagy, but had little effect on protein levels of HSC70 (Figure 6H,I). We further found that LAMP2A protein levels significantly increased, though LC3B II and HSC70 levels showed no significant changes in the brain lysates of PS19;*59^+/−^* mice compared to those of PS19 mice (Figure 6J,K). TMEM59 downregulation also increased LAMP2A levels in HEK293T cells (Figure S7A,B in supporting information). On the other hand, TMEM59 overexpression decreased LAMP2A protein levels and increased LC3B‐II levels without affecting HSC70 protein levels in HEK293T cells (Figure S7C,D).

We then studied how TMEM59 affects LAMP2A protein levels. When TMEM59 was downregulated in HEK293T cells, we found that LAMP2A protein degradation was markedly reduced (Figure 6L,M), suggesting that TMEM59 modulates LAMP2A protein degradation. Finally, we found that LAMP2A overexpression reduced tau phosphorylation at sites S202/T205 and S396 without affecting total tau levels (Figure S7E,F). These results indicate that TMEM59 haploinsufficiency enhances the CMA activity by increasing LAMP2A protein levels, thereby promoting the degradation of phosphorylated tau.

## 4 DISCUSSION

Tauopathy includes a series of clinically heterogeneous neurodegenerative diseases that share a common tau pathology.^1^, ^2^, ^3^, ^4^ AD is the most common type of tauopathy. In addition to the tau pathology, AD has another major pathological hallmark, Aβ deposition in the brain.^29^, ^30^ We and others previously found that TMEM59 deficiency could attenuate disease‐like phenotypes in 5xFAD and APP/PS1 mice, two AD model mice exhibiting Aβ pathology.^13^, ^14^ Herein, we found that TMEM59 protein levels were increased in the brains of AD patients and PS19 mice at pathological stages. These results are consistent with previous findings showing that *TMEM59* gene expression is increased and *TMEM59* promoter DNA methylation is decreased in AD patients,^31^, ^32^ and that TMEM59 protein levels are increased in the brains of 5xFAD mice at pathological stages.^14^ Importantly, we further found that TMEM59 haploinsufficiency attenuated disease‐like phenotypes, including cognitive deficits, neurodegeneration, synapse dysfunction, gliosis, neuroinflammation, and tau pathology in PS19 mice. These findings demonstrate the correlation between TMEM59 and AD and suggest that TMEM59 may become a therapeutic target for AD and other tauopathic disorders.

Hyperphosphorylated tau is a major form of tau pathology.^1^, ^2^, ^3^ We found that TMEM59 deficiency reduced phosphorylated tau levels without affecting total tau levels. Because tau is phosphorylated by kinases such as GSK3β and CDK5‐P35^33^, ^34^, ^35^, ^36^ and dephosphorylated by phosphatases such as PP1α, PP2A, and PP2B,^37^, ^38^ we examined but found that TMEM59 deficiency had no effects on protein levels of GSK3β and CDK5‐P35, PP1α, PP2A, and PP2B, and on general kinase and phosphatase activities, implying that TMEM59 deficiency reduces phosphorylated tau levels through other mechanisms.

CMA is one type of autophagy that degrades proteins to maintain cellular proteostasis.^5^, ^6^, ^7^ During CMA, the chaperone protein HSC70 recruits substrate proteins to the lysosomal membrane, and then LAMP2A mediates the translocation of CMA substrates into the lysosomal lumen for degradation.^5^, ^8^ We found that TMEM59 interacted with both HSC70 and LAMP2A. TMEM59 deficiency promoted protein levels of LAMP2A, the interaction between LAMP2A and HSC70, and the CMA activity, whereas TMEM59 overexpression had the opposite effects. Moreover, TMEM59 downregulation reduces LAMP2A degradation. Because LAMP2A is the rate‐limiting component of CMA,^5^, ^8^ our findings indicate that TMEM59 deficiency elevates LAMP2A protein levels by inhibiting its degradation, thereby enhancing the CMA activity. Several studies have demonstrated that CMA can degrade neurodegeneration‐related proteins, including tau.^5^, ^6^ A previous study revealed that LAMP2A deficiency accelerated tau phosphorylation and aggregation without affecting total tau levels in an AD mouse model.^5^ Therefore, our findings suggest that TMEM59 deficiency reduces tau phosphorylation by increasing the CMA activity to promote pathologic tau degradation, rather than modulating tau phosphorylation/dephosphorylation processes.

It is reported that TMEM59 can interact with ATG16L1 and facilitate macroautophagy, another type of autophagy.^10^, ^11^, ^12^ Studies in cultured cells found a bi‐directional cross‐talk between CMA and macroautophagy, with macroautophagy deficiency inducing CMA and CMA blockade upregulating macroautophagy.^39^, ^40^ However, the bi‐directional cross‐talk between CMA and macroautophagy may be cell type specific, as one study found that although macroautophagy downregulation resulted in enhanced CMA activity, CMA blockade failed to activate macroautophagy in photoreceptor cells.^41^ Herein, we found that TMEM59 overexpression not only reduced LAMP2A protein levels and the CMA activity but also promoted LC3B II levels indicative of macroautophagy in HEK293T cells. Consistently, we observed increased LAMP2A protein levels and decreased LC3B II levels in primary neurons derived from PS19;*59^+/−^* mice compared to those from PS19 mice. However, only LAMP2A protein levels significantly increased, whereas LC3B II levels showed no significant decrease in the brain tissues of PS19;*59^+/−^* mice compared to those of PS19 mice. One possibility for these inconsistent results is that the existence of some non‐cellular components in the brain tissues masks the change of LC3B II levels. Alternatively, a bi‐directional cross‐talk between TMEM59‐mediated CMA and macroautophagy may be cell specific, and a heterogeneity of neural cell types in the brain masks the change of LC3B II levels.

Moreover, we noticed that HSC70 mRNA levels were increased in the brain of PS19 mice and decreased upon TMEM59 haploinsufficiency. However, HSC70 protein levels were not significantly altered. HSC70 has diverse functions in many cellular pathways besides CMA, such as the unfolded protein response and chaperone.^42^ Therefore, HSC70 protein levels may be saturated in the cell, and TMEM59 deficiency may not be enough to perturb HSC70 protein levels.

In addition to HSC70, our RNA‐seq and mass spectrometry results identified many other genes/proteins involved in misfolded protein responses, implying that these pathways may also participate in TMEM59 deficiency‐mediated protection in tauopathy. Although we found that TMEM59 haploinsufficiency had no effect on key proteins mediating the UPR response, further scrutiny of this and other pathways is required.

We previously found that TMEM59 interacted with TREM2 to modulate TREM2‐dependent microglial activities. Because TREM2 mutations and functional changes are closely related to AD,^12^, ^43^ this raises the possibility that TMEM59 deficiency attenuates microgliosis and neuroinflammation in PS19 mice through directly affecting TREM2. However, our previous study showed that TMEM59 overexpression had no effect on TREM2 protein stability.^12^ Another study also reported that TMEM59 knockout did not alter the protein levels of TREM2.^13^ Therefore, it is probable that attenuated microgliosis and neuroinflammation in PS19;*59^+/−^* mice results indirectly from pathologic tau reduction, other than directly from TREM2 function change.

In conclusion, our study not only demonstrates that TMEM59 plays an important role in tauopathy but also identifies a crucial function of TMEM59 in binding HSC70 and LAMP2A to regulate the CMA activity. These findings suggest that targeting TMEM59 and its downstream CMA activity may provide a novel therapeutic strategy for tauopathy.

## CONFLICT OF INTEREST STATEMENT

The authors have declared that no conflicts of interest exists. Author disclosures are available in the supporting information.

## CONSENT STATEMENT

Human brain tissue lysates were requested through MTA from National Developmental and Functional Human Brain Bank, Chinese Academy of Medical Sciences, National Health and Disease Human Brain Tissue Resource Center, and Brain Bank and Neurodegenerative Disease Research Center in China. Consent was not necessary for the authors.

## Supporting information



Supporting Information

Supporting Information

Supporting Information

## Data Availability

All data are available upon reasonable request.
