# Supplementary material for: TMEM59 deficiency activates chaperone‐mediated autophagy and ameliorates disease‐like pathologies in tauopathy model mice
Source: Alzheimers Dement. 2025 Jun 23;21(6):e70369. doi: 10.1002/alz.70369 (PMC12185246; doi:10.1002/alz.70369)
Supplement: Supplementary file 1 — Supporting Information [file ALZ-21-e70369-s002.pdf]

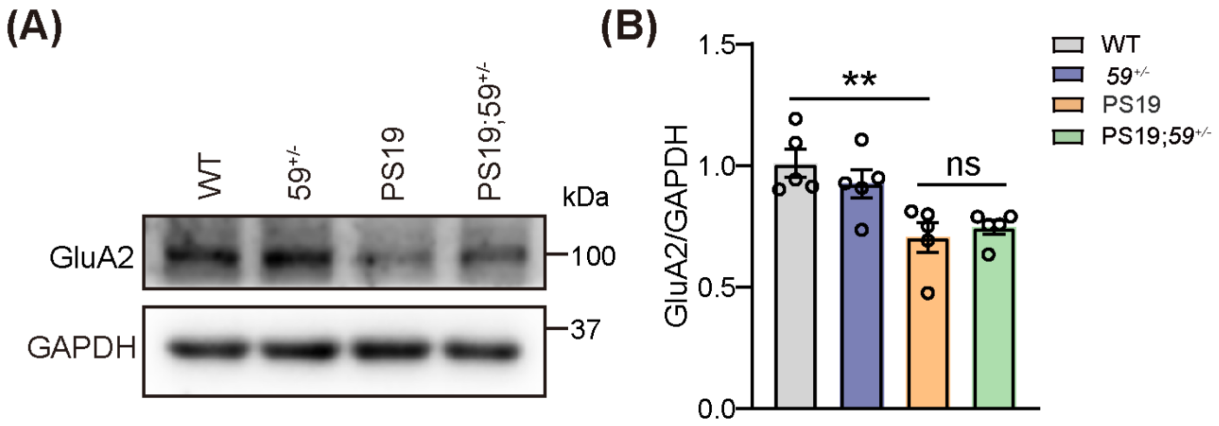

FIGURE S1. TMEM59 haploinsufficiency does not alter the levels of GluA2 proteins. (A and B) Western blotting of GluA2 in 9.5- to 10-month-old mouse brain lysates (A) and their quantitative comparison (B). n = 5 mice per group. Data represent mean ± SEM. *P* values were determined by one-way ANOVA with Tukey's post hoc analysis. ns, not significant; $**P < 0.01$.

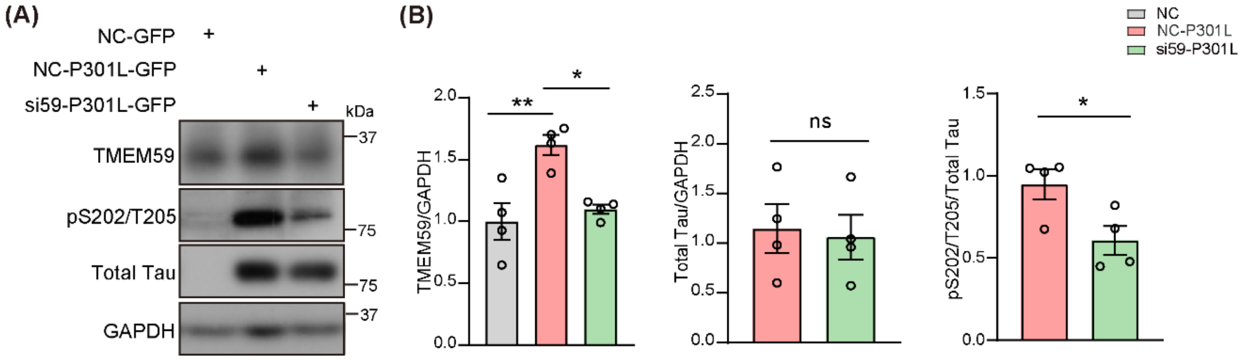

FIGURE S2. TMEM59 downregulation reduces tau hyperphosphorylation *in vitro*. (A and B) HEK293T cells expressing tau P301L-GFP were transfected with TMEM59-targeting siRNA (si59) or control siRNA (NC). Equal amounts of protein lysates were subjected to western blotting (A) and quantitative comparisons (B) of TMEM59, total tau, and tau phosphorylated at sites Ser202/Thr205 (pS202/T205). n = 4 per group. Data represent mean ± SEM. P values were determined by one-way ANOVA with Tukey's post hoc analysis (for TMEM59) and unpaired t test (for total tau and tau pS202/T205). ns, not significant; *$P < 0.05$; **$P < 0.01$.

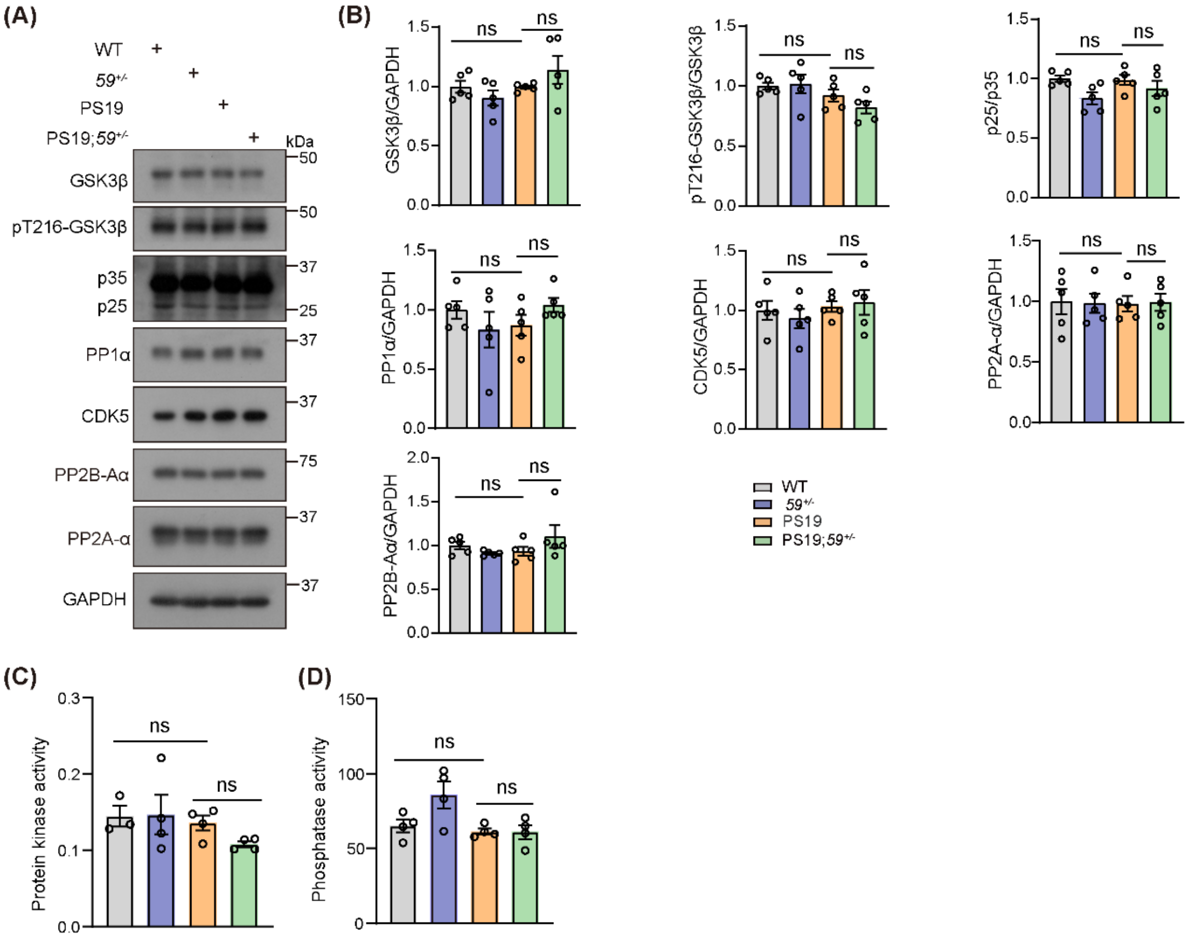

FIGURE S3. *Tmem59* haploinsufficiency has no effect on protein kinase activity and phosphatase activity in PS19 mice. (A and B) Western blotting of GS3Kβ, pT216-GS3Kβ, p25/p35, PP1α, CDK5, PP2A-α, and PP2B-Aα in 9.5- to 10-month-old mouse brain lysates (A) and their quantitative comparisons (B). $n = 5$ mice per group. (C and D) The protein kinase activity (C) and phosphatase activity (D) in 9.5- to 10-month-old mouse brain lysates were assayed for comparisons. $n = 3\text{-}4$ mice per group. Data represent mean ± SEM. P values were determined by one-way ANOVA with Tukey's post hoc analysis. ns, not significant.

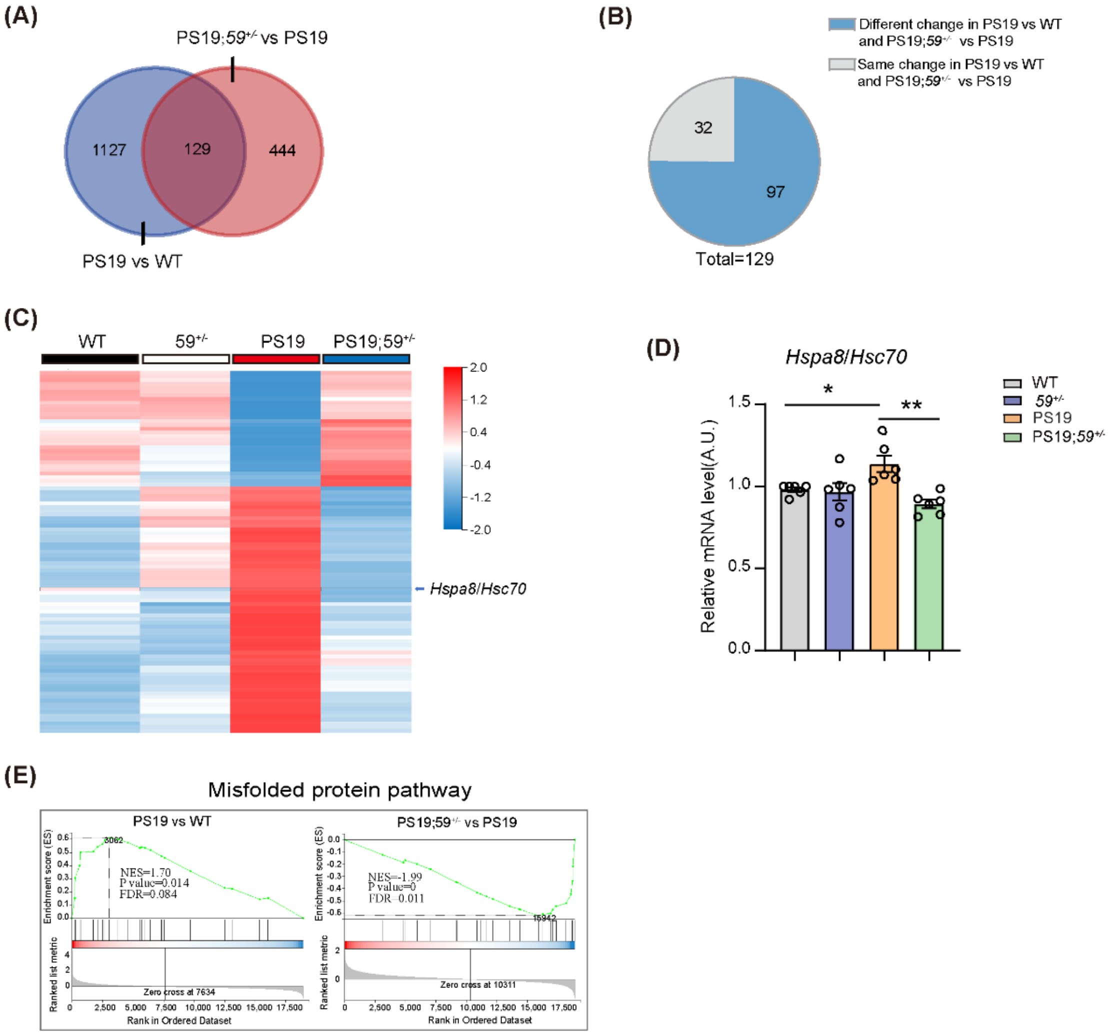

FIGURE S4. Analysis of RNA-seq results. (A) Venn diagram analysis for DEGs found in the PS19 vs WT group and in the PS19;*59*$^{+/-}$ vs PS19 group. (B) Comparisons of the change directions of DEGs between the PS19 vs WT group and the PS19;*59*$^{+/-}$ vs PS19 group. (C) The unsupervised clustering gene expression heatmap for the 97 DEGs with different change directions between the PS19 vs WT group and the PS19;*59*$^{+/-}$ vs PS19 group. Higher abundant proteins are shown in red and lower abundant proteins are in blue, with a Z-score of protein intensity displayed in color. (D) qRT-PCR analysis of *Hspa8/Hsc70* mRNA levels in the mouse brain. n = 6 mice per group. (E) Gene set enrichment analysis (GSEA) of the misfolded protein pathway in PS19 vs WT (left) and

PS19;*59*$^{+/-}$ vs PS19 (right) groups. Data represent mean ± SEM. *P* values were determined by one-way ANOVA with Tukey's post hoc analysis. *$P < 0.05$; **$P < 0.01$.

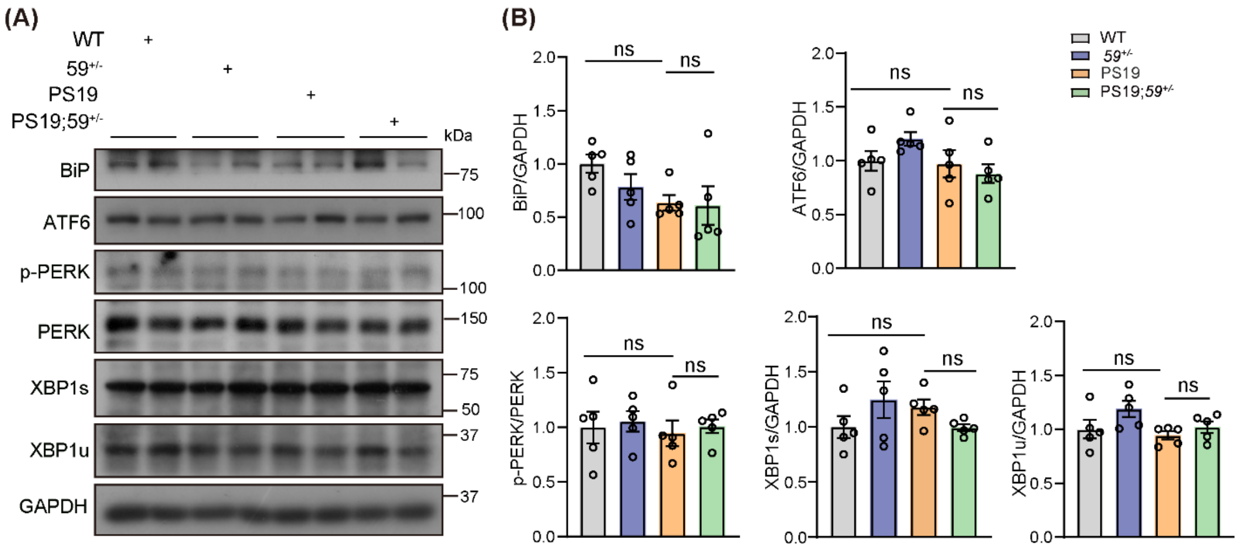

FIGURE S5. *Tmem59* haploinsufficiency has no effect on UPR-related protein levels. (A and B) Western blotting (A) and quantitative comparisons (B) of BiP, ATF6, p-PERK, spliced XBP1 (XBP1s), and unspliced XBP1 (XBP1u) protein levels in 9.5- to 10-month-old mouse brain lysates. n = 5 mice per group. Data represent mean ± SEM. P values were determined by one-way ANOVA with Tukey's post hoc analysis. ns, not significant.

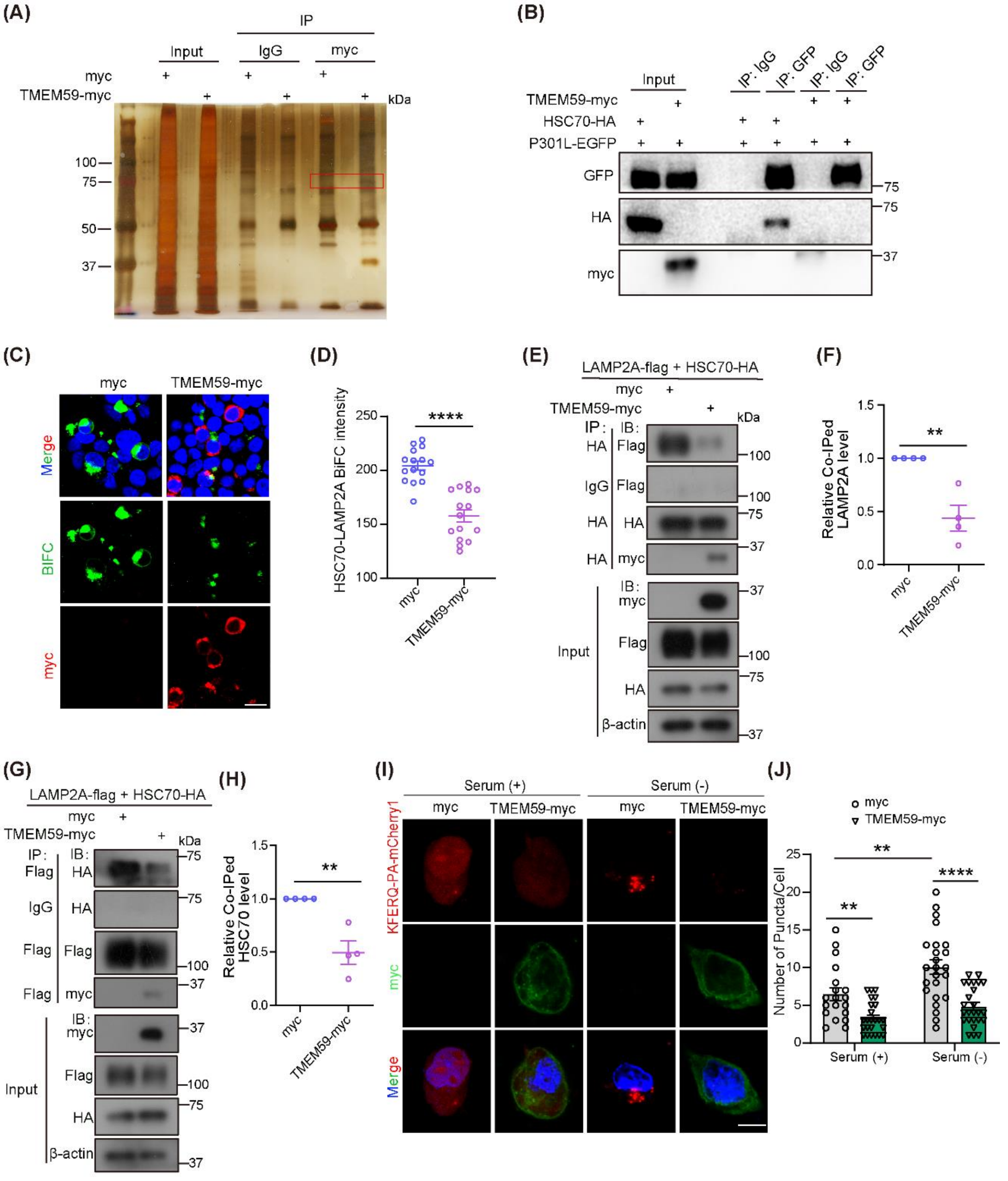

FIGURE S6. TMEM59 overexpression inhibits the CMA activity. (A) HEK293T cells were transfected with TMEM59-myc or myc control. Equal amounts of protein lysates were subjected to immunoprecipitation (IP) with IgG or an anti-myc antibody. Immunoprecipitated proteins were

run on SDS-PAGE gel and stained with silver nitrate. One band of about 70 kDa (highlighted in red box) was identified specifically in cells transfected with TMEM59-myc and immunoprecipitated with the anti-myc antibody. (B) Tau P301L-EGFP was co-transfected with HSC70-HA or TMEM59-myc in HEK293T cells. Equal amounts of protein lysates were immunoprecipitated (IP) with IgG or an anti-GFP antibody and immunoblotted with anti-GFP, anti-HA, and anti-myc antibodies. (C and D) HEK293T cells were first co-transfected with HSC70-VN173 and LAMP2A-VC155, and then transfected with TMEM59-myc or myc control. Cells were immunostained with an anti-myc antibody and an appropriate fluorescence-labelled secondary antibody, stained with DAPI, and observed under a confocal microscope (C). Green color representing the HSC70-LAMP2A interaction was quantified for comparison (D). Red color indicates TMEM59-myc. Scale bar, 20 μm. n = 15 cells per group. (E-H) HEK293T cells were first co-transfected with HSC70-HA and LAMP2A-flag, and then transfected with TMEM59-myc or myc control. Equal amounts of protein lysates were immunoprecipitated (IP) with IgG and an anti-HA antibody (E), or with IgG and an anti-flag antibody (G). Immunoprecipitated proteins were subjected to immunoblotting (IB) with indicated antibodies (E, G). LAMP2A-flag levels immunoprecipitated by the anti-HA antibody in (E) were normalized to input levels for comparison (F). HSC70-HA levels immunoprecipitated by the anti-flag antibody in (G) were normalized to input levels for comparison (H). n = 4 per group. (I and J) HEK293T cells were first transfected with KFERQ-PA-mCherry1, and then transfected with TMEM59-myc or myc control. After photoconverting, cells were maintained in media supplemented with (+) or without (-) serum for 16 h. Cells were then immunostained with an anti-myc antibody and an appropriate

fluorescence-labelled secondary antibody, stained with DAPI, and observed under a confocal microscope (I). Red puncta in cells were quantified for comparisons (J). Scale bar, 10 μm. $n = 19 - 25$ cells per group. Data represent mean ± SEM. P values were determined by unpaired t test in (D, F, and H), and two-way ANOVA with Tukey's post hoc analysis in (J). $^{**}P < 0.01$; $^{****}P < 0.0001$.

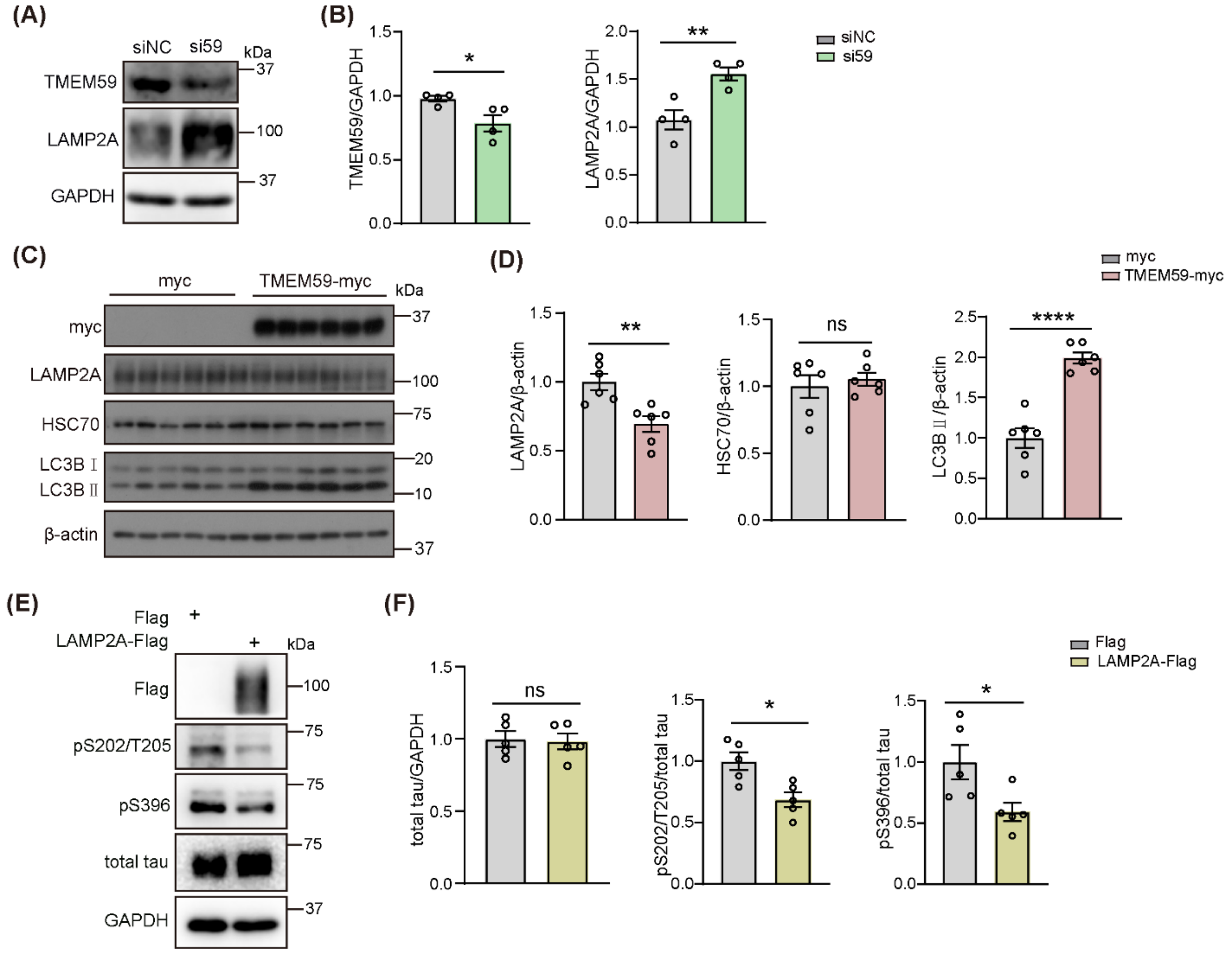

FIGURE S7. TMEM59 modulates LAMP2A protein levels that affect tau phosphorylation. (A and B) HEK293T cells were transfected with TMEM59-targeting siRNA (si59) or control siRNA (siNC). Equal amounts of protein lysates were subjected to western blotting (A) and quantitative comparisons (B) of TMEM59 and LAMP2A. n = 4 per group. (C and D) HEK293T cells were transfected with TMEM59-myc or myc control. Equal amounts of protein lysates were subjected to western blotting (C) and quantitative comparisons (D) of LAMP2A, HSC70, and LC3B-II. n = 6 per group. (E and F) HEK293T cells expressing tau P301L-myc were transfected with LAMP2A-Flag or Flag control. Equal amounts of protein lysates were subjected to western

blotting (E) and quantitative comparisons (F) of total tau and tau phosphorylated at sites Ser202/Thr205 (pS202/T205) and Ser396 (pS396). $n = 5$ per group. Data represent mean ± SEM. P values were determined by unpaired t-test. ns, not significant; $^{*}P < 0.05$; $^{**}P < 0.01$; $^{****}P < 0.0001$.
